# Mass Spectrometry-Based Glycoproteomics and Prostate Cancer

**DOI:** 10.3390/ijms22105222

**Published:** 2021-05-14

**Authors:** Caterina Gabriele, Licia E. Prestagiacomo, Giovanni Cuda, Marco Gaspari

**Affiliations:** Research Centre for Advanced Biochemistry and Molecular Biology, Department of Experimental and Clinical Medicine, Magna Graecia University of Catanzaro, 88100 Catanzaro, Italy; cuda@unicz.it (G.C.); gaspari@unicz.it (M.G.)

**Keywords:** SPEG, TiO_2_, PSA, glycocapture, biomarker discovery, protein glycosylation

## Abstract

Aberrant glycosylation has long been known to be associated with cancer, since it is involved in key mechanisms such as tumour onset, development and progression. This review will focus on protein glycosylation studies in cells, tissue, urine and serum in the context of prostate cancer. A dedicated section will cover the glycoforms of prostate specific antigen, the molecule that, despite some important limitations, is routinely tested for helping prostate cancer diagnosis. Our aim is to provide readers with an overview of mass spectrometry-based glycoproteomics of prostate cancer. From this perspective, the first part of this review will illustrate the main strategies for glycopeptide enrichment and mass spectrometric analysis. The molecular information obtained by glycoproteomic analysis performed by mass spectrometry has led to new insights into the mechanism linking aberrant glycosylation to cancer cell proliferation, migration and immunoescape.

## 1. Introduction

Prostate cancer (PCa) is the most common and the second most lethal cancer in men in the United States with an estimated 248,530 new cases and 34,130 deaths in the current year [1]. To date, PCa detection is based on digital rectal exam (DRE) and on serum dosage of PSA but the use of PSA is affected by its low specificity and sensitivity and by its inability to discriminate between aggressive (AG) and indolent tumours [2,3]. In fact, PCa treatment poses some concerns about its clinical behaviour, being this tumour silent and often non-aggressive (NAG) in some individuals (low-risk PCa) and AG with early progression and metastasis onset in others (high-risk PCa) [4]. Consequently, the discovery of new diagnostic and prognostic biomarkers of PCa is of pivotal importance.

Most of the known biomarkers are proteins. Thus, in recent years, proteomics has become a supporting science for clinical research. Proteomic analysis, in fact, allows to shed light on the alterations correlated with pathological events by characterizing and quantifying proteins and their post-translational modifications (PTMs) [5]. Among protein PTMs, glycosylation is the more widespread modification, and it is implicated in several biological processes [6]. About 250–500 genes are responsible for protein glycosylation [7], determining the generation of 3000 different glycans structures; the microheterogeneity is further complicated by several possible combinations of different glycan linkages, generating a high number of variants. Glycans can be attached to the polypeptide chain in two manners: O-glycosylation on serine or threonine amino acid residues and N-glycosylation on asparagine residue with consensus sequence asparagine-X-Serine/Threonine (where X is any amino acid except proline). The glycoproteome is not static but it is extremely dynamic and dependent on cell type, tissue differentiation and disease status [8]. The observed changes are not the direct product of gene expression but are the result of an intricate balance between different elements: the correct synthesis and function of enzymes involved in the process, the availability of sugars and the activity of the necessary enzymes for sugar metabolism [9]. When the harmony between these different elements is perturbed, as it happens during malignant transformation, altered glycoform expression (over-expression, under-expression, neo-expression) can be encountered [10]. Glycoproteins are usually secreted or membrane proteins. As a consequence, glycoproteome alterations greatly impact on crucial cellular processes such as cell signalling, invasion, immune modulation, angiogenesis and cell-cell interaction [11,12].

In the light of the wealth of information that can be collected through glycoprotein analysis and considering the current lack of a specific biomarker for PCa, the application of glycoproteomics analysis could be a winning path to delineate a clearer picture of this disorder. To date, glycoproteomics of PCa is an expanding branch of research because it offers the possibility of identifying proteins with a central role in PCa, thus unveiling aspects that straightforward whole proteome analysis could not detect. Moreover, the study of glycoproteins in PCa is important because the prostate gland produces secreted proteins, and these are usually glycosylated. When the prostate gland undergoes a malignant transformation its architecture is altered with evident changes (in size and shape) and progressive epithelium reduction; these modifications lead to important effects on the secretory pathways and the altered and aberrant glycosylation represents a probable consequence of all the perturbations occurring after tumour onset [13]. Over the years, many efforts were focused on the study of prostate specific antigen (PSA) and its glycosylations. Notably, several studies have demonstrated that the integration of PSA glycosylation analysis with serum dosage of PSA could help distinguishing PCa from benign prostate hyperplasia (BPH). Moreover, PSA from PCa patients shows increased levels of fucosylation [14] and α2,3-linked sialic acid [15] compared to PSA from BPH patients. The increment of fucosylation in PCa patients seems to be correlated with the up-regulation of fucosyltransferases, the enzymes involved in fucosylation process; for instance, the fucosyltransferase 6 (FUT6) is over-expressed in patients with bone metastasis [16].

**Figure 1:**
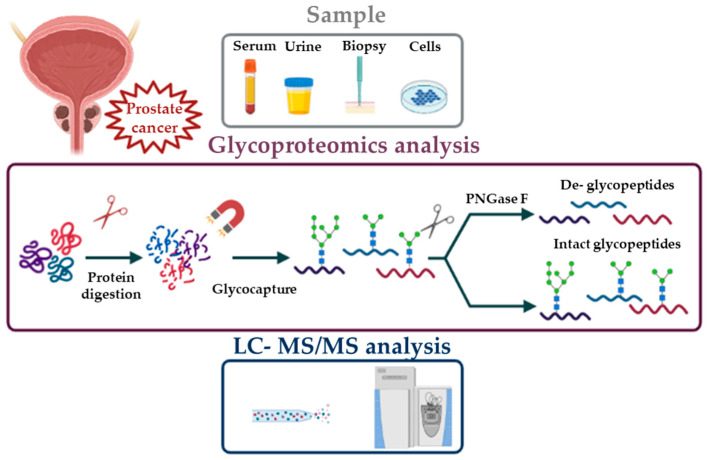
Bottom-up approach.

Many goals in the glycoproteomic field have been achieved by the development and improvement of several techniques such as mass spectrometry (MS). MS based approaches have the great advantage of potentially allowing the full characterization of glycoproteins through the analysis of amino acid sequence, the identification of glycosylation sites and the study of attached sugars. However, the simultaneous analysis and characterization of the different components by MS is a challenging task [17]. For this reason, the choice of the proper workflow for sample preparation and analysis is of primary importance. To date, there is no universal method for glycoproteomic analysis; in particular, two different approaches can be used: top-down and bottom-up. Both strategies are based on the isolation of glycoproteins from the whole proteome. The top-down approach consists in the direct analysis of intact proteins by MS to obtain information about protein sequence, sugar composition and glycosylation sites. The drawback of this experimental design is that its applicability is limited to simple mixtures of small glycoproteins [18]. Definitely, the most frequently used approach for glycoproteomics is bottom-up, which is based on the MS analysis of proteolytic peptides. After enzymatic digestion and glycopeptide enrichment (see below), two different paths can be undertaken: the first is based on the chemical or enzymatic removal of glycans from glycopeptides [19] followed by mass spectrometric analysis of formerly glycosylated peptides; the second strategy is characterized by the direct analysis of intact glycopeptides by MS [20] (Figure 1).

These two different bottom-up strategies provide complementary information for glycoprotein characterization. In fact, the removal of attached glycans produces peptides which are more suitable for MS/MS analysis, but it only provides information, about the identity of proteins and their glycosylation sites. Conversely, intact glycopeptide analysis, despite a greater analytical complexity, provides information about attached glycans.

**Figure 2:**
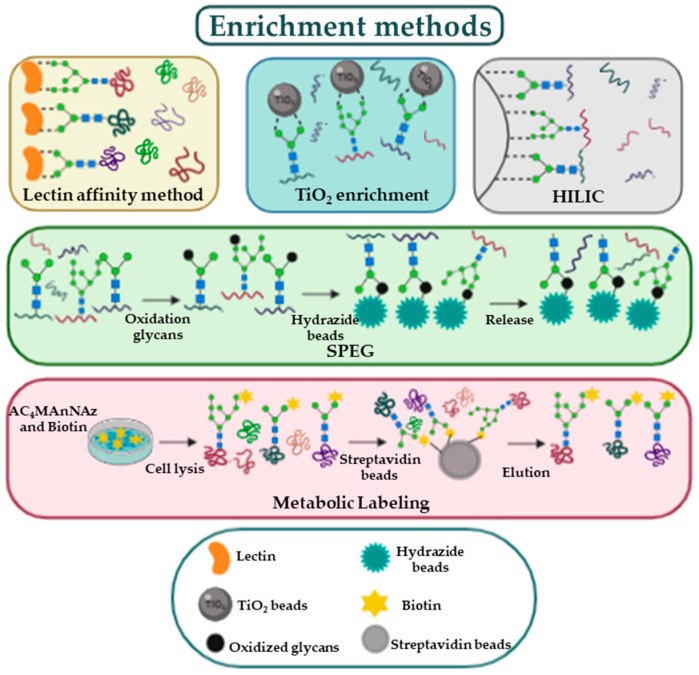
This figure is a simple representation of the enrichment methods.

An additional challenge in glycoproteomics is the low abundance of many glycoproteins; this problem is solved by utilizing enrichment methods (Figure 2).

Both glycoproteins and glycopeptides can be separated from non-glycosylated proteins using lectins [21]; lectins are carbohydrate-binding proteins exhibiting characteristic glycan-binding specificity; some can bind specific glycans while other have a wider range of recognized glycans [22]. The advantage of lectin affinity enrichment is the reversibility of the bond between lectins and the sugars, which allows to characterize the glycan originally present on the glycoproteins. Another enrichment method is solid phase extraction of glycopeptides (SPEG) [23]. In this method, exploiting hydrazide chemistry, the oxidation of carbohydrate moieties is the necessary step to covalently attach the glycopeptides on a solid support. After enrichment, formerly N-glycosylated peptides are removed from the solid support through Peptide:N-Glycosidase F (PNGase F). De-glycopeptides are then characterized by MS. In 2007 Larsen et al. have developed a protocol utilizing titanium dioxide (TiO_2_) for capturing sialic acid-containing glycopeptides; this method is based on the high affinity of sialic acids for TiO_2_ beads in a specific buffer [24]. All enrichment methods, since they involve several steps, constitute a potential source of error. For this reason, the quality of results can be improved by the use of labelling protocols [25]. For instance, a labelling approach, known as metabolic oligosaccharide engineering, uses synthetic monosaccharides to label glycoproteins directly in cells and in experimental animals. Among available molecules, the azido monosaccharides are often used in these experiments because they are small and not present in cells [26]; glycoproteins incorporating the functional groups are separated from the other cellular components by the use of a reaction with affinity probes [25].The isolated glycopeptides can be identified and quantified by MS analysis obtaining a relative quantification between the different conditions.

Finally, an important challenge to improve glycoproteomics analysis could be the development of a database, a precious box in which the glycoforms associated to a specific pathology are collected. A potential source of information is UniCarbKB, a database where the sugar structures and the characterized glycosylation sites are collected; these data are freely available; everyone can explore the glycoforms associated with specific disorders [27]. In the future, the implementation of new software will probably allow the realization of the “Human glycome project” [28].

The progress made is there for all to see, but much more needs to be done to improve both glycoproteomic workflows and software for data interpretation. Despite the gaps to fill, MS has demonstrated to be a great support for clinical research contributing to delineate a deep proteomic map for several samples. The big challenge of the future will be to detect proteins indicating early tissue alterations in easily collectable biofluids, thus limiting the use of invasive exams.

In this context, we will discuss the most promising works related to MS-based glycoproteomic studies on PCa. In the text, to allow a more fluent reading, and since MS actually identifies gene products, protein names are replaced with gene names.

## 2. Glycoproteomics of Cells and Tissues

Glycoproteomic analysis of cells and tissues is a charming approach to investigate the mechanisms involved in PCa development and progression, giving insights into signalling pathways that trigger the tumour onset and promote metastasization. We will start by reporting studies performed on cell cultures, which highlighted how differences in the glycoprotein profiles could be caused by the alterations in the expression of the enzymes involved in the glycosylation process.

LNCaP (androgens dependent) and PC3 cells (androgens independent) were used by Shah et al. [29] to analyse glycoproteins structure, glycosylation sites and bound sugars; this work characterized for the first time the glycoproteome of these cell lines. Peptides were labelled by the iTRAQ reagent (isobaric Tags for Relative and Absolute Quantification) and fractionated to analyse the whole proteome and to perform the quantitative analysis on the intact glycopeptides. An aliquot of the labelled peptide mixture was enriched by SPEG and analysed after PNGase treatment. Overall, 8063 peptides and 653 N-glycoproteins were identified, 176 of which were found differentially abundant between LNCaP and PC3 cells. The comparison between the differentially expressed glycoproteins (176) and global proteome showed that for 156 glycoproteins (88.11%) the observed differences were attributable to changes in the amount of the corresponding protein, while for 21 glycoproteins (11.9%) these variations were ascribed to differential glycosylation. For this reason, the observed changes in the two cell lines could reflect a variation in protein abundance or a change in enzyme expression involved in the glycosylation process. The whole proteome analysis highlighted that two fucosyltransferases (FUT8 and FUT 11) were up-regulated in PC3 cells. This differential enzymatic expression could be the cause of the observed changes in the glycan structure of some glycoproteins. Notably, APMAP, HSP90B1, PSAP showed an increase in fucosylation levels and a decrease in oligomannose content in PC3 cells respect to LNCap cells. The function of FUT8 was furtherly investigated by Wang et al. [30]. They showed that FUT8 inhibition (by siRNA technology) in PC3 cells decreased cell motility: a crucial process in neoplastic evolution. Moreover, using tissue microarrays the authors affirmed that FUT8 was elevated in metastatic tissue vs. normal tissue and that over-expression of FUT8 was associated with high Gleason score; they concluded that FUT 8 could be used as potential prognostic biomarker.

The same group has published a follow-up study focused on protein fucosylation in PCa cell lines [31]. After comparing six different lectins known to have specificity for fucosylated peptides, they adopted the best possible protocol for glycopeptide enrichment, consisting in protein digestion, fucosylated glycopeptide capture by *Lens culinaris agglutinin* (LAC), further purification of glycopeptides by a hydrophilic interaction chromatography-based mechanism, and glycopeptide quantification based on isobaric mass tagging of the intact glycopeptides. The protocol was applied to the study of four different PCa cell lines, namely two NAG types (LNCaP, LAPC4) and two more AG types (PCA3, DU145) and allowed the quantification of 973 glycopeptides (on 252 distinct glycoproteins), among which 51 resulted significantly increased in AG cell lines by partial least squares-discriminant analysis (OPLS-DA). Among the differential peptides, 13 belonged to three proteins: ITGA2, ITGA3 and ITGB1. The authors postulate that increased fucosylation, for which the fucosidase FUT8 seems to be responsible, might activate integrin-mediated cell migration and signalling. In order to investigate further a possible role of FUT8 in PCa development, and in particular in the development of castration resistant prostate cancer (CRPC), the same group performed and additional proteomic study [32]. They implemented a comprehensive comparative proteomic analysis (whole proteome, phosphoproteome and glycoproteome) on: (i) LNCaP cells overexpressing FUT8, (ii) LNCaP control cells and (iii) PC3 cells. They detected increased levels of EGFR in LNCaP-FUT8 cells with respect to wild type cells. The authors suggested that increased levels of EGFR were possibly due to increased stability of the protein itself via increased levels of core-fucosylation of EGFR by FUT8. EGFR phosphopeptide levels resulted also increased in LNCaP-FUT8 cells, together with phosphopeptides of several proteins linked to EGFR downstream signalling, such as GRBs, SRC, MEKK1, MEK1, JAK1, and PKC. Overexpression of FUT8 would also suppress PSA production by cancer cells and promote resistance to gefitinib-induced cell death. The protein FUT8 was investigated further in vivo, by comparing FUT8 expression in tumour sections of xenograft mice which were subjected to orchiectomy *versus* non-castrated mice (control group). Immunohistochemistry data showed induction of FUT8 under androgen-restricted conditions and overall increase of core-fucosylation in cancer tissue. The information reported above brought light on an important aspect: the study of differentially expressed glycoproteins can guide the identification of altered activity of enzymes involved in the glycosylation process providing a crucial information on the tumour environment. A better knowledge of cancer perturbations is of pivotal importance for the development of a targeted therapeutic strategy.

Glycosylation is abundant on proteins localized on the cell surface. Since these proteins are in direct contact with the extracellular microenvironment, they could be involved in mechanisms that support tumour onset and progression. Some studies have been focusing on the analysis of sialylated surface glycoproteins, since sialylation may correlate with cancer progression, possibly favouring immunoescape [33]. An elegant workflow, specifically targeted at surface/extracellular sialoglycoproteins based on bioorthogonal labelling, has been described in a few studies [34,35]. Cells were labelled with peracetylated N-acetylmannosamine (Ac_4_ManNAc), which would be converted in the corresponding sialic acid derivative, and finally incorporated into the glycoproteins in vitro. The reaction with an enrichment probe (generally a terminal alkyne-biotin) would allow the isolation of the labelled sialoglycoproteins by affinity capture.

Yang et al. have applied the workflow described above to the analysis of cell surface sialoglycoproteins in non-metastatic (N2) and highly metastatic (ML2) PC3 cancer cell lines [36]. Proteomic analysis of the captured sialoglycoproteins was achieved by SDS-PAGE separation at the protein level followed by in-gel digestion and nLC-MS/MS. After enrichment they have cumulatively identified 538 proteins (372 from ML2 and 324 from N2 cells). The efficacy of the enrichment method was demonstrated by the fact that 26% and 30% of the identified proteins were, respectively, secreted proteins and cell-surface proteins. Moreover, a semi-quantitative comparison by spectral counting has detected, in ML2 cells, the overexpression of several proteins involved in growth process (SLC38A2, JUP, PTPN1, POSTN, CALR, BSG, PNN, CDCP1, POLR1E, COL6A1), invasion (BSG, POSTN, GPI, CALR, LRRC15, MARCKS), migration and cell mobility (JUP, PTPN1, NPC1, SLC38A2, CAP1), all processes linked to malignant transformation.

Protein sialylation was also studied by Spiciarich et al. [35] using a similar approach to that used by Yang et al. based on metabolic labelling with AC_4_ManAz and covalent immobilization on beads by click chemistry. Compared to the previously described work, in Spiciarich et al. normal (n = 8) and PCa (n = 8) tissues were analysed using a more complex model. In total, the authors have identified 972 proteins, 68% of which were either membrane-bound or secreted. The comparative analysis between cancerous and normal tissue resulted in 24 proteins found increased in PCa by a factor higher than 4-fold (VDAC1, DPP2, EZRI, GDF15, FOLH, CATB, AMPN), though quantitative analysis was performed using a low precision approach (spectral counting). Interestingly, the authors found an average 22-fold increase of VDAC1 in PCa *versus* normal tissue. This observation could be justified by the increase in concentration of the protein itself, or by an increase of its glycosylation levels. The role of VDAC1 in PCa was confirmed by another study on PC-3 cells, where the silencing of VDAC1 expression led to the inhibition of both cell proliferation and tumour growth in xenografts [37]. The overall results of this small-scale study confirmed that cancer disease has a specific sialylation profile.

Discriminating between aggressive and indolent behaviour of PCa is of pivotal importance. Chen et al. have searched for glycoproteins associated to AG PCa by applying SPEG technology to optimal cutting temperature (OTC)-embedded tissue slices [38]. This work was an extension of a proof of principle paper published by the same group two years earlier [39], in which the authors described the methodology and applied it to a limited number of cases (eight in total). In this follow-up work, their discovery sample set consisted of 31 specimens from NAG and 24 from AG PCa. LC-MS/MS analysis identified 350 formerly N-glycosylated peptides belonging to 242 glycoproteins. Among the identified proteins, using a low-precision quantitative approach based on spectral counting, they revealed 17 differentially abundant proteins. Among them, COMP, CTSL, APMAP, AOC3, POSTN, CSPG2. In order to perform ELISA validation on a second sample set (27 NAG, 20 AG), they chose the most promising candidates by an interesting prioritization approach based on literature analysis. Proteins previously associated to PCa and to cancer aggressiveness were selected for validation. This second set of experiments confirmed the overexpression of COMP and POSTN and the decreased expression of CSPG2 in AG compared to NAG PCa.

Biomarkers of tumour aggressiveness were also investigated by Liu et al. They carried out a comparative analysis between normal prostate (n = 10), NAG (n = 24), AG (n = 16) and metastatic (n = 25) cancer tissues [40]. After protein extraction and digestion, glycopeptides were isolated by SPEG, treated with PNGase F and analysed by sequential window acquisition of all theoretical mass spectra (SWATH-MS). A spectral library, for protein identification and quantification, was previously generated by the analysis of pooled samples and synthetic reference peptides, resulting in a total of 2188 N-glycosites and 897 N-glycoproteins. The increased library space has allowed the identification of, on average, 1430 N-glycosites per analysis. Compared to previous studies, these results largely expanded the number of identified N-glycosites. Moreover, statistical analysis has led to a list of 220 proteins with altered expression profile among the different groups. Extensive literature search revealed that the majority of these regulated proteins was previously correlated to PCa and/or associated with general tumour aggressiveness. Besides, 50 out of 220 proteins were significantly differentially expressed between AG and NAG. Glycoproteomic analysis has revealed that NAAA and PTK7 were associated with AG cancer. The validation on tissue microarray has indicated that these two candidates could be used to discriminate between AG and NAG phenotypes. Their protein list was very interesting because 17 proteins were previously identified in human serum by selected reaction monitoring (SRM) and 7 of these (TIMP1, ATRN, ASPN, CADM1, BTD, HYOU1, NCAM1) were considered potential diagnostic PCa biomarkers [41]. Notably, more than 75% of identified proteins in this tissue study are found in human plasma at low concentrations (<100 ng/mL) but, nonetheless, still detectable by MS. Thus, the obtained candidates are suitable for further validation studies. For what was mentioned above, this study represents an excellent starting point for the identification of novel biomarkers able to discriminate between normal and different cancer stages: AG, NAG and metastatic.

In order to evaluate if PCa progression was accompanied by glycoproteome perturbations, Kawahara et al. [42] performed a glycoproteomic analysis on 5 tissues from patients with BPH and 50 from PCa patients. The latter were furtherly divided in five different groups (n = 10 per group) based on Gleason score. Briefly, after protein digestion, peptides were labelled by Tandem Mass Tags (TMT) and N-glycopeptides were enriched by ZIC-HILIC (zwitterionic Hydrophilic Interaction Liquid Chromatography) glycopeptide enrichment. Non-modified peptides (in the flow-through), intact glycopeptides (from ZIC-HILIC enrichment), and de-N-glycopeptides (from ZIC-HILIC enrichment after PNGase F treatment) were all subjected to high-pH reversed phase solid phase-extraction (SPE) fractionation followed by LC-MS/MS analysis; glycans released by PNGase treatment were also analysed. Through this approach it was possible to identify approximately 500 N-glycoproteins and 200 O-glycoproteins, obtaining a deep map of the tissue glycoproteome and, as a consequence, increasing the probability of detecting differences between BPH and PCa glycoproteome, possibly highlighting glycoforms correlated with tumour progression. Mostly, glycan analysis permitted to evaluate the alterations in the abundance profile of some glycosylation types, highlighting changes between PCa groups and BPH (*t*-test, *p* > 0.05). In particular, the groups with a low grade PCa were characterized by elevated levels of paucimannosidic-(glycans with low percentage of mannose) and monoantennary-complex-type N-glycans respect to BPH group. High grade PCa, instead, was richer in highly branched complex-type N-glycans, while oligomannosidic-, hybrid- and biantennary complex-type N-glycan showed decreased levels when compared to BPH group. Moreover, to untangle the rich web of molecular information, the identified proteins were compared with the proteins of prostatic tissue, the proteins of bone marrow and the extracellular matrix (ECM) proteins, all information enclosed in Protein Atlas. These data demonstrated changes (increased oligomannosylation) correlated with the tumour stage for glycoproteins of tissue and ECM origin. In addition, the identified paucimannosidic glycans belonged to glycoproteins of immune cells, further indication of the extreme complexity of tumour environment. This study clearly demonstrates that, in the actual scenario, the complete picture of the glycoproteome is only achievable using very complex workflows.

PCa, like several neoplastic diseases, is characterized by dynamic events, release mediators and additional components, such as immune cells, playing a key role in the determination of tumour fate. Tumour microenvironment significantly contributes to cancer proliferation, metastasis, and resistance to therapy. In particular, it is known that metastatic castration-resistant prostate cancer tumours (mCRPC) are often enriched in M2 macrophages; these immune cells promote a permissive cell growth environment through the secretion of cytokines, matrix degrading enzymes, angiogenic factors and multiple growth factors. Based on these information, Zarif et al. aimed at identifying M2 enriched surface glycoproteins that may serve as therapeutic targets [43]. Glycoproteomics analysis was performed on human CD14+ monocytes and homogeneous macrophage populations. After SPEG enrichment and de-glycosylation by PNGase F, N-linked glycopeptides were analysed by LC-MS/MS. Overall, 176 unique glycopeptides from 114 proteins with 1% FDR were identified. Label free semi-quantitative analysis based on spectral count demonstrated that MRC1, CTSL, ITGA3, LGMN and SLC9A7 were enriched in M2. Moreover, as a further confirmation of the permissive role performed by M2 in cancer development, they discovered that homogeneous populations of M2 macrophages secreted anti-inflammatory cytokines such as IL-10 and, more importantly, the angiogenic factor VEGF-A. Then, the confirmation of M2 infiltration in mCRPC was carried out by flow cytometry, using MRC1 and M2 macrophage scavenger receptor CD163, whereas M2 infiltration in bone metastasis (a common site of mCRPC metastasis) was demonstrated by immunohistochemistry, by staining for MRC1. In conclusion, this study demonstrated M2 macrophage infiltration in human mCRPC. Besides, some surface glycoproteins in these immune cells were indicated as enriched. These proteins, in particular MRC1, seem to be specific of macrophages M2 and, for this reason, could be potential therapeutic targets.

Some of the most relevant findings obtained in the studies described in this paragraph are summarized in Table 1.

## 3. Glycoproteomics of Biofluids

After tumour onset, tissue architecture is modified; this morphologic change leads to the release into systemic circulation of molecular cues highlighting tumour presence. Proteomics and, in particular, glycoproteomics of body fluids, allowing to retrieve information about membrane and/or secreted proteins, represents a rich source of precious information potentially useful for monitoring cancer onset and/or progression.

### 3.1. Urine

The analysis of the urinary glycoproteome is of great relevance for the characterization of binding sites, structures of the carbohydrate chains and glycoproteins released by prostate gland. Unlike biopsies and blood, the use of urine samples for PCa biomarker discovery has distinct advantages. Primarily, sample collection is not invasive and urinary proteins do not degrade quickly after collection [44]. Furthermore, due to the anatomic proximity of the prostate to the bladder and urethra, urine may contain prostatic secretions and exfoliated prostate epithelial cells empowering the identification of glycoproteins reflecting prostate health status.

Kawahara et al. have analysed urinary glycoproteins using a single urine sample, from a healthy man, to describe N-linked glycosylation sites and glycopeptides with low molecular weight [45]. Briefly, urine samples collected in two different days were centrifuged and 20 mL of supernatant were concentrated on filters with a 10 kDa cut-off (Millipore, Billerica, MA). The flow–through was subjected to hydrophilic-lipophilic-balanced (HLB) SPE to characterize low molecular weight endogenous glycopeptides, while proteins captured on the filter were digested with trypsin. Glycopeptide enrichment was performed by HILIC protocol; an aliquot of eluate was deglycosylated by PNGase F. The spectral analysis of intact glycopeptides was conducted with Byonic software while the identification of proteins and glycosylation sites was performed by MaxQuant. In total, 256 glycoproteins with 472 unique N-glycosylation sites were characterized and 90 glycoproteins with 202 unique N-glycosylation sites were identified by analysing the flow-through. This complex workflow led to a thorough characterization of the urinary glycoproteome, though it has to be considered a proof of principle study since it was applied to a single sample from a healthy donor.

In order to evaluate whether urine was a reliable source of prostate specific proteins and, in particular, of PCa-associated glycoproteins, a glycoproteomic analysis on urine and serum samples from AG and NAG PCa patients was carried out from Jia et al. [46] The *primum movens* of this study was to determine which of these biological samples could be a better source of information about the prostatic patho-physiological state. For this study, 40 urine samples from PCa patients and 167 serum samples (n = 119 PCa, n = 48 without PCa) were collected. N-linked glycosite containing peptides were isolated by SPEG and released from hydrazide beads by PNGase F. After enrichment and de-glycosylation, peptides from sample pools from either urine or serum were separated in 24 fractions and subjected to LC-MS/MS analysis. In total, 2923 and 2472 formerly N-glycosylated peptides were identified in serum and urine, respectively. The authors showed that 40% of tissue glycopeptides identified in a previous study [40] were also detectable in urine, whereas only 13% was detectable in serum, suggesting that urine, in view of its proximity to the prostate, may be a more favourable source of prostate-derived proteins than serum. In the same work, the authors performed label free quantitative glycoproteomic analysis on an additional sample set consisting of 20 urine samples from patients suffering from either AG (n = 10) or NAG (n = 10) disease. The relative amount of five glycoproteins, namely PTK7, ICOSLG, AZGP1, FBN1 and GLG1 was significantly decreased in urine samples from patients with AG disease.

In a valuable study of Kawahara et al., glycoproteins in urine samples collected from BPH and PCa patients were compared [47]. In brief, after protein digestion, peptides were TMT-labelled before enrichment of glycopeptides by TiO_2_ and HILIC protocols. The analysis was performed by MS both on intact N- and O-glycopeptides and on formerly N-glycosylated peptides and desialo-O-glycopeptides obtained by treatment with, respectively, PNGase F and sialidase A. Results showed 56 distinct intact N-glycopeptides able to fully discriminate between BPH and PCa groups. Interestingly, the abundance of both formerly N-glycosylated peptides and corresponding proteins was not able to separate the two groups, highlighting that the use of specific glycoforms provided a more effective PCa-specific signature. However, the reliability of the conclusions that were drawn from this study is undermined by the very limited extent of the sample set (5 PCa and 4 BPH).

Urinary expressed prostatic secretions, or EPS-urine, is a sample collected after DRE. Compared to urine, EPS-urine is enriched in prostate specific proteins. For example, Vermassen et al. showed that average total PSA urinary concentration (tPSA) before and after DRE was, respectively, 75.5 µg/L and 13,030 µg/L [48]. Dong et al. analysed EPS-urines from 74 and 68 patients with AG PCa and NAG PCa, respectively; they aimed to detect differentially expressed glycoproteins among the two groups by data independent analysis (DIA) [49]. Briefly, the digestion of 500 µL of EPS-urine was performed by automated tryptic digestion on C4-tips (Lys-C 1 h and trypsin 6 h) [50]; after digestion, N-glycopeptides were isolated by automated C18/MAX-tip method followed by de-glycosylation with PNGase F and peptide purification by C18 StageTip [51]. To build the spectral library for DIA analysis, 142 EPS-urine samples were pooled, and N-glycopeptides were separated in 8 distinct fractions by basic reversed-phase liquid chromatography before LC-MS/MS analysis in data-dependent mode. DIA data analysis revealed that, out of a total of 1289 unique glycopeptides detected, belonging to 594 glycoproteins, 79 glycopeptides showed differential abundance between NAG e AG groups. Among the 79 glycopeptides, 54 showed a fold change of at least 1.5 with an estimated FDR of 0.25. The results include glycopeptides belonging to proteins already known to be related to PCa, such as ACCP, CD63 (decreased in AG disease) and DSC2, LOX, LRG1, CLU, SERPINA1, ORM1 (increased in AG disease). In addition, using the glycopeptides cited above, the discrimination power of different biomarker panels, was evaluated: ACCP in combination with serum PSA alone, or ACCP in combination with serum PSA together with each one of the following candidates: CLU, LOX, SERPINA1, ORM1. The best model was the combination of ACCP, CLU and serum PSA, which provided an AUC of 0.86, with specificity of 50% and sensitivity of 95%. These results were tested in two validation cohorts. In comparison with the discovery phase some candidates such as DSC2 and LRG1 showed a lower discrimination power, while others confirmed their capacity to discriminate between AG and NAG (Table 2) allowing to delineate a panel with the best candidates.

The obtained results seem promising but need further validation with a larger cohort. However, the experimental design of this study encloses several points of strength: (i) the choice of a sample, EPS-urine, enriched of prostatic derived proteins; (ii) the use of an automated system for sample processing increasing the protocol reproducibility; (iii) the implementation of DIA, a sensitive analytical method allowing to detect low abundance proteins.

Some of the most relevant findings obtained in the comparative analyses described in this paragraph are summarized in Table 3.

### 3.2. Serum

Blood is a potential gold mine for the discovery of novel candidate biomarkers as it keeps information about all body systems. In its route through the body, blood takes close contact with all tissues and organs, acquiring valuable information about the individual’s health. Tissue-derived proteins are released in the blood circulation by secretion or leakage and are ultimately diluted to low concentrations, which typically lie in the ng/mL range. Consequently, tissue proteins are only a negligible fraction of the whole blood protein content, thus their detection by proteomic techniques is very challenging, ultimately complicating the use of serum as the sample of choice for the discovery of new cancer biomarkers.

A valuable attempt to overcome these issues for PCa biomarker discovery was made by Cima et al., in 2011 [41]. They developed a two-stage strategy articulated in an initial phase of discovery applied to a mouse model of PCa progression and then in a second phase of validation on human serum and tissues. Briefly, in the first stage, PCa candidate biomarkers were discovered by enriching N-linked glycopeptides through SPEG to detect and quantify differentially expressed glycoproteins in the prostate tissue (n = 8) and sera (n = 8) of Pten cKO mice compared to control animals. In the second stage, selected candidates were measured in serum by targeted proteomics and ELISA to evaluate the association of PTEN-inactivation in human PCa with a specific serum signature (n = 143, 66 BPH and 77 PCa). As a result, they identified a five protein signature comprising GALNTL4, FN, AZGP1, BGN and ECM1 that predicted patients having tumours with a Gleason score <7 or ≥7 with an AUC of 0.788; and a four protein signature comprising ASPN, CTSD, HYOU1 and OLFM4 discriminating between BPH and PCa groups with an AUC of 0.726. Intriguingly, the combination of the four protein signature and PSA resulted in an AUC of 0.840. Subsequently, Kalin et al., assessed if the quantitative proteome alterations following PTEN loss could enclose prognostic biomarkers in mCRPC [52]. In particular, this study combined the use of the candidates derived from the cancer-genetics guided model proposed by Cima et al., with previously validated biomarkers used in prognostic nomograms. Formerly N-glycosylated proteins were quantified by either mass-spectrometry based targeted proteomics or ELISA in sera of 57 mCRPC patients processed as described in the aforementioned study. The results showed a five-factor predictor, comprising THBS1, CRP, PVLR1, EFNA5 and MME having an accuracy of 96% and 94% in predicting 12- and 24-months survival, respectively.

In 2015 Thomas et al., in an attempt to discover a biomarker signature able to distinguish AG PCa from indolent, NAG PCa, developed a “tier 2” multiplexed targeted MS assays for the quantification of N-glycopeptides in serum by parallel reaction monitoring (PRM) [53]. PRM assays, being usually based on the use of isotopically labelled internal standards, are characterized by high precision. The assay relies on parallel precursor isolation and fragmentation of target ions followed by a single MS/MS scan at high resolution. This scan mode is suitable for targeted verification of selected candidates found in discovery experiments. In this case, the starting point was a list of 377 glycopeptides which were considered potential targets of interest in two previous works on PCa tissue published by the same authors [38,39]. N-linked glycopeptides were enriched by SPEG followed by the specific release of formerly N-linked glycosylated peptides by PNGase. Forty-three N-linked glycopeptides were selected for PRM quantification based on their detectability in serum, and 41 could be reproducibly quantified in 75 serum samples (n = 25 AG, n = 25 NAG, n = 25 without PCa). Among the 41 assay targets, only 4 N-linked glycosite-containing peptides showed significantly higher levels (*p* < 0.05) in serum from the NAG vs. AG patient groups: AFNSTLPTMAQMEK (CD44); EEQFNSTFR (IGHG2); GAFISNFSMTVDGK (ITIH2); and INNTHALVSLLQNLNK (CDH13). Despite of the appreciable investigation strategy, this study showed no significant differences between PCa (AG, NAG) and non-PCa groups.

In 2018 Totten et al., performed a quantitative glycoproteomic analysis using multi-lectin affinity chromatography (M-LAC) to compare the circulating levels of proteins and their glycoforms in sera of BPH and PCa patients (n = 10 PCa, n = 7 BPH) [54]. This study was focused on glycosylation alterations previously reported to be aberrant in PCa: the increase in glycan branching as well as the increase of fucosylation and sialylation. To this end, *Aleuria aurantia lectin* (AAL) were used to capture intact core-fucosylated proteins and *Phaseolus vulgaris leucoagglutinin*/*erythroagglutinin* (PHA-L/E) were used to capture glycoproteins containing highly branched glycans. Serum samples (BPH and PCa) were subjected to immunodepletion of the top 14 most abundant proteins; nondepleted proteins were alkylated using heavy ^13^C-acrylamide. Proteins from a sample pool were derivatized by light ^12^C-acrylamide in order to represent a reference sample for relative quantification. All heavy/light mixes were subjected to affinity capture by either AAL or PHA-L/E lectins. Bound (AAL, PHA-L/E) and unbound (UNB) fractions were subjected to reversed-phase chromatography at the intact protein level (n = 3 × 13 fractions per sample); finally, protein fractions were digested by trypsin, and resulting peptides were analysed by LC-MS/MS. Relative quantification was based solely on acrylamide-labelled peptides. As a result, the authors observed alterations in circulating levels of proteins both at the global level and in specific glycoforms (thus specifically enriched in either AAL or PHA-L/E fractions). Both PCa and BPH groups showed quite similar protein expression patterns confirming the difficulty to identify glycoproteins able to efficiently differentiate between the two groups. Despite a high level of similarity in glycoprotein content, some significant differences were observed. Global levels of CD5L, CFP, C8A, BST1, and C7 proteins were significantly increased in the PCa samples. Besides, glycoform-specific alterations between BPH and PCa were identified among proteins such as CD163, C4A ATRN in the PHA-L/E fraction, and C4BPB and AZGP1 glycoforms in the AAL fraction, all of which were over-expressed in the PCa group.

In 2018, Sajic et al. employed a cross-tumour comparison to determine the molecular similarities and differences within the plasma/serum proteome in different types of localized-stage carcinoma (colorectal, pancreatic, lung, prostate, and ovarian) [55]. Blood samples (n = 284) were analysed with a proteomic workflow combining N-glycosite enrichment and SWATH MS. In particular, to increase the coverage of low abundant tissue derived proteins, N-glycosylated peptides were selectively enriched by SPEG and analysed after de-glycosylation by PNGase F. The results of this multi-cancer comparison revealed that localized tumours display “specific biomarkers” for each individual cancer type as well as “common biomarkers” which derive from a systemic response to cancer. This study showed that FBN1, THBS1 and ITIH3, involved in platelet activation, signalling and aggregation, change across different tumour types and appear to be sensitive to general blood cancer biology, while Pregnancy zone protein (PZP), as previously reported in the literature, confirmed to be significantly changed in the serum of PCa patients, thus emerging as a potential specific candidate biomarker of PCa [42].

Gabriele et al., in 2018 developed a high-throughput protocol which allowed a consistent detection of low abundance proteins in serum via enrichment of sialylated peptides by TiO_2_ beads, de-glycosylation by PNGase F and nLC-MS/MS analysis either in data-dependent or in targeted mode [56]. In a first step, a peptide library of over 700 formerly N-glycosylated peptides was generated by data-dependent LC-MS/MS analysis of glycopeptides from serum pools of both PCa patients and healthy controls (6 pool in total). Then, 16 medium to low abundance proteins (DSG2, IL6ST, LAMP2, PLXNB2, GOLM1, CTSD, TIMP1, PTPRF, IGFBP3, ASPN, POSTN, APMAP, LAMP1, LCN2, PIGR, PEDF) were selected and quantified in duplicates by SRM on a cohort of 54 patients (PCa = 24 and BPH = 31). Four formerly N-glycosylated peptides belonging to four different proteins APMAP, POSTN, CTSD and LAMP2 were found significantly increased in PCa sera compared to the control group.

Results of these studies are summarized in Table 4. Though Table 4 only contains a selection of DAPs found in each work, it is evident that there is limited overlap between the different studies. For example, only two proteins (AZGP1 and POSTN) were identified as DAPs in three different studies. The low incidence of common discoveries is possibly due to multiple reasons. Probably, the most important factor is that the focus of the studies was different, varying from comparing AG to NAG disease, to discriminating between PCa and BPH, to comparing the glycoproteomic profile of several cancer types. Besides, sample preparation and MS instrumentation employed were different, resulting in large variations in terms of proteome coverage and in the type of analytes detected (deglycopeptides, intact glycopeptides, glycoproteins).

**Figure 3:**
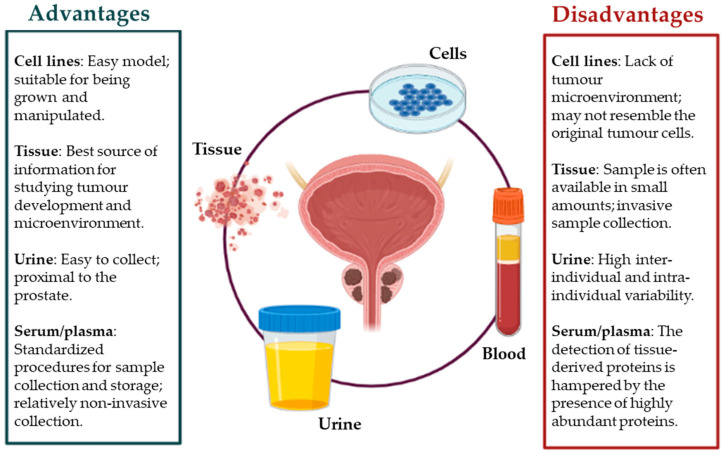
Advantages and disadvantages for each sample type.

Overall, we summarize the principal advantages and disadvantages for each sample type mentioned above (Figure 3).

## 4. Prostate Specific Antigen

Since being introduced in the clinic in the mid-1980s, PSA blood test has been widely used for PCa early detection in association with DRE.

**Figure 4:**
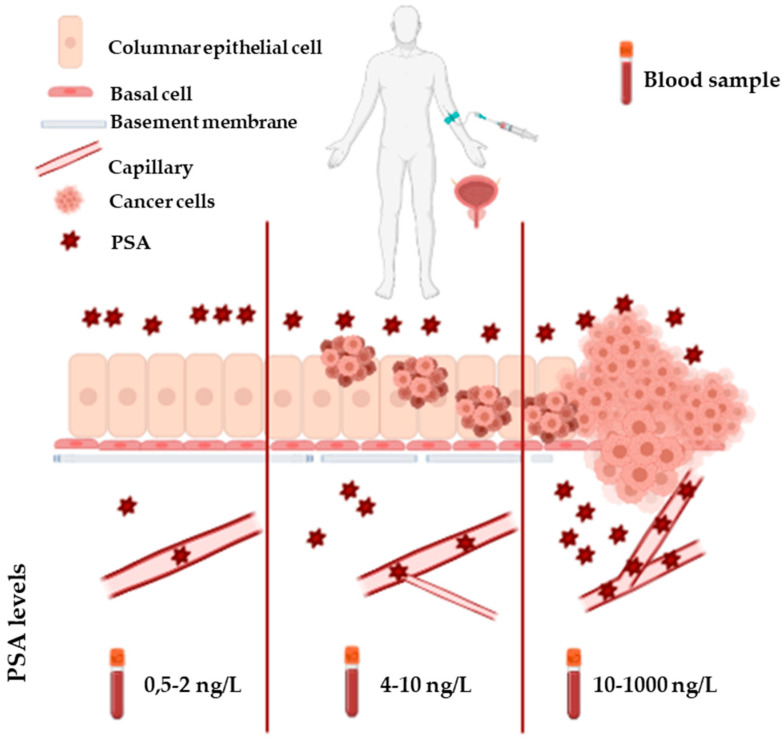
This figure shows the correlation between the alteration of tissue architecture and the increase of serum levels of PSA in PCa.

PSA, an androgen-regulated serine protease, is a 28.7 kD glycoprotein exclusively produced by the prostatic gland. It belongs to the kallikrein family and is encoded by the KLK3 gene. PSA is synthesized by the columnar epithelial cells as a 261-amino acid (aa) prepro-protein having a 17-aa signal sequence. After cleavage, the signal sequence is released into the prostatic ducts to generate an inactive 244–amino acid precursor protein (proPSA). In the prostatic ducts, proPSA is activated by enzymatic cleavage (7 N-terminal aa) in PSA: a 237-aa protein with a single glycosylation site at Asn-45 (Asn-69 in preproPSA). PSA is a major protein in semen, where its function is to cleave semenogelins in the seminal coagulum. However, small amounts of this glycoprotein are also found in the blood circulation of healthy individuals as consequence of its spread through some anatomic barriers such as the basement membrane, the stromal layer, and the walls of blood and lymphatic capillaries [57,58]. High PSA levels in serum, as a result of the loss of the architecture of the prostate gland and the disruption of basal cells and basement membrane by tumour cells are found in PCa patients [59] (Figure 4).

This occurrence represents the rationale of PSA blood testing. However, significant limitations plague PSA blood screening. In fact, increased levels of PSA in blood are not cancer-specific; other conditions can raise PSA blood levels such as benign prostate hyperplasia (BPH), prostatitis, or manipulations of the prostate (e.g., bicycling or catheterization). Moreover, PSA testing lacks sufficient sensitivity, because serum levels of PSA do not necessarily increase in presence of advanced PCa. Another issue with PSA blood test is “the diagnostic grey zone” (men with blood concentrations of PSA between 4–10 ng/mL) in which only the 25% of patients have PCa, leading to the execution of many unnecessary biopsies [60,61]. Importantly, this test does not differentiate between indolent and aggressive forms of PCa. As a consequence, the clinical implementation of this screening has led to reduced incidence of advanced disease and mortality, but also to overdiagnosis and overtreatment [62]. Thus, several efforts have been made to improve the diagnostic and prognostic power of PSA, such as: (i) normalizing PSA on the basis of the prostate gland volume (PSA density), (ii) monitoring the kinetics of PSA in serum (PSA velocity, PSA doubling time), (iii) measuring multiple molecular traits (e.g., free and complexed PSA). In particular, the ratio of free to total PSA (%-free PSA) has been shown to decrease in PCa compared to BPH and has been approved by FDA for use in patients which fall in the diagnostic grey zone. Further improvements have been made by the introduction of tests measuring different isoforms of PSA, namely the Prostate Health Index (PHI) and the 4Kscore. PHI combines measurements of free, total, and (-2)pro-PSA (an isoform preferentially produced by cancer cells) into a single score, while the 4Kscore measures a panel of four kallikreins: free PSA, total PSA, intact PSA, and kallikrein-like peptidase 2 (hK2). Both tests have outperformed %-free PSA in detecting PCa [60].

Thus, a viable way to increase the specificity of this test and a possible solution for all these issues could be the characterization and quantification of specific PSA isoforms. In view of what was mentioned above and on the basis of new evidence linking cancer development to protein glycosylation alterations, many studies have been focusing on the study of PSA glycoisomers.

In the PCa glycoproteomics scenario, MS has emerged as a powerful tool for glycoprotein characterization and quantification. The great potential of this technique derives from its ability to provide both quantitative and structural information such as glycosylation position, glycan composition and importantly quantitative differences between different conditions. However, to improve the characterization of glycoproteins and, in particular, of PSA glycoforms, there is still the need to couple MS analysis with upstream separation techniques, such as liquid chromatography or capillary electrophoresis. This strategy, greatly improves the resolution and the sensitivity of the analysis but also impacts on sample throughput [63]. Given the above, the use of matrix-assisted laser desorption/ionization (MALDI-MS) represents an attractive “separation-free” alternative. A recent study about male infertility has allowed the quantification of 44 PSA glycopeptides through a high-throughput and easy to use platform by MALDI-MS. PSA was captured from seminal plasma by bead-based affinity purification and subjected to tryptic digestion. The stabilization of α2,6- and α2,3-sialylated isomers was achieved by a two-step amidation reaction; then glycopeptides were enriched by HILIC SPE and analysed by MALDI-MS. This quick workflow could represent a good starting point for future PSA glycoforms characterizations in PCa [64].

The principal mass spectrometric studies about PSA were mostly focused on the characterization of its glycan structures using commercially available PSA or cell lines, while only few studies have explored the differences of PSA glycoforms between healthy and cancer state in clinical samples.

PSA has different degrees of sialylation and this results in heterogeneity of its charge. In the literature, two isoforms of PSA are known: the major isoform consisting of over 90% of PSA having an isoelectric point of 6.9, and the minor one, with an isoelectric point of 7.2 (PSA high isoform, PSAH). The principal characterization of the glycan structures associated with the two isoforms of PSA was made as part of the 2012 ABRF Glycoprotein Research Group study [65]. This interlaboratory study was focused on the characterization of the two glycoforms of PSA commercially available (PSA and PSAH) by MS. The aim of this study was to evaluate state of the art mass spectrometry-based methods in the determination of differences in glycoprotein structures between the two isoforms. The choice of PSA, with its characteristics of low-molecular weight protein bearing a single glycosylation site, allowed the implementation of multiple analytical strategies including intact protein analysis (top-down approach), analysis of glycopeptides (bottom-up approach) and analysis of glycans released after PNGase treatment. Each participating laboratory (22 in total) produced a list of N-glycan compositions with indication of their corresponding relative intensities for both PSA and PSAH isoforms. Statistical and comparative analyses were used to identify a consensus from the resulting data. A consensus cluster representing 17 of the 22 participating laboratories, was identified, producing consistent results for differential N-glycan composition between the two PSA isoforms. Notably, 61 N-glycan were identified, 8 of which differed significantly in abundance between the two PSA samples. The results demonstrated a major presence of disialylated and fucosylated structures in PSAH than in PSA. As part of this study, Behnken et al., characterized PSA and PSAH isoforms by a top-down approach, coupling high resolution LC-MS and bioinformatics (mathematical deconvolution of isotopically resolved ion patterns and database analysis) [66]. This “plug and play” strategy allowed the identification of 38 glycoforms. A further characterization of the 2 isoforms of PSA was made by Song et al., which previously took part in the ABRF study. Tryptic digestion of the two separate PSA isoforms produced 3 peptide backbones: NKSVILLGR, AVCGGVLVHPQWVLTAAHCIRNK, and AVCGGVLVHPQWVLTAAHCIRNKSVILLGR. Fifty-six N-glycans were associated to PSA, whereas 57 N-glycans were observed in the case of PSAH; the majority of the observed glycans were identified on the NKSVILLGR backbone. Interestingly, 3 sulphated/phosphorylated glycopeptides were identified. The glycan structures were quantified by spectral counting and the results demonstrated a good correlation with the 2012 ABRF [67].

Another PSA isoform which has attracted interest as potentially PCa-specific is the one bearing α2,3-sialic acid. Below, some examples of promising studies focusing on α2,3-sialylated isoform of PSA are reported. The following studies are not based on mass spectrometric analysis. In 2014, Yoneyama et al. developed a magnetic microbead-based immunoassay measuring the amount of α2,3-sialic acid-linked PSA in serum in a training set of 100 samples (non-PCA = 50 and PCa = 50) and then in a validation set of 314 samples (Non-PCa = 176 and PCa = 138) [68]. The diagnostic accuracy of this assay was compared with that of conventional PSA and %fPSA. The results showed a sensitivity of 95.0% and 90.6% and a specificity of 72.0% and 64.2% for the diagnosis of PCa in the training and validation sets, respectively. Moreover, in the validation study, the AUC for the detection of PCa bearing α2,3-sialic acid resulted to be significantly higher than that obtained by PSA or %fPSA (α2,3-sialic acid = 0.84, %fPSA = 0.60, PSA 0.61). Another paradigmatic example of the importance of PSA glycoforms in support of future clinical decisions is the study of Ferrer-Betallè et al. They compared the performance of PHI with a glycoform assay measuring the α2,3-sialic acid percentage of PSA in serum to discriminate between BPH and PCa patients (BPH = 29 and low-risk = 7, intermediate risk = 21, high-risk = 22 PCa) [69]. Briefly, total PSA was immunopurified and then applied to a lectin chromatography using *sambucus nigra* (SNA)-agarose lectin, which binds to α2,6-sialylated glycoconjugates allowing the separation of α2,3-sialylated from α2,6-sialylated PSA glycoforms. Eluted unbound and bound chromatographic fractions were collected and the quantification of PSA in these fractions was made using electrochemiluminescence technology (the bound fraction corresponded to α2,6-sialic acid PSA while unbound fraction corresponded to α2,3-sialic acid PSA). As a result, the % of α2,3-sialic acid outperformed PHI in separating high-risk PCa from the other groups (AUC of 0.971 vs. 0.840). The combination of both markers increased the AUC up to 0.985 resulting in 100% sensitivity and 94.7% specificity to differentiate high-risk PCa from the other low and intermediate-risk PCa and BPH patients. These results are promising but require further validation studies.

Kammeijer et al. developed a method based on capillary electrophoresis–mass spectrometry (CE–MS) allowing the selective analysis of α2,3- and α2,6-sialylated glycopeptides [70]. This separation platform was applied to the analysis of tryptic glycopeptides of commercially available PSA after glycan enrichment by cotton HILIC SPE. Seventy-five glycopeptides, all attached to the tryptic dipeptide “N69K” were detected, many of which were biantennary structures harbouring 2 terminal sialic acids. The results showed a good separation of α2,3- and α2,6-linked isomers. However, the principal drawback of this set up was the high amount of PSA required for the analysis (1 ng of PSA was injected and analysed), a quantity more readily available in urine samples than in serum. Indeed, this analytical strategy was used to develop a PSA Glycomics Assay (PGA), for the differentiation of α2,6- and α2,3-sialylated isomers of PSA in urine [71]. After affinity purification and tryptic digestion of PSA, samples were analysed by CE-ESI-MS (capillary electrophoresis-electrospray ionization coupled to mass spectrometry). This strategy was applied on 23 urine samples (PCA = 13, non-PCA = 10) from patients suspected of PCa. After tryptic digestion, 0.5 µL of urine deriving from each patient were used to create a sample pool, subsequently used to characterize PSA glycopeptides, as previously done with commercially available PSA. A total of 67 N-glycopeptides, all attached to the dipeptide “N69K” were identified. This assay demonstrated a good intra-day and inter-day variability which were below 3% and 7% (RSD), respectively: well below the assessed biological variation (RSD of 50%). Despite the low technical variability of PGA, no significant differences were detected among the two groups, probably due to limited sample size. Van der Burgt et al., in a proof of principle study, showed a partial separation of sialic acid linkage-specific isoforms of PSA [72]. This exploratory study coupled the use of hydrophilic interaction liquid chromatography (HILIC) with targeted quantitative MS for the analysis of PSA glycopeptides obtained after tryptic or ArgC digestion of commercially available human PSA. However, this strategy resulted only in a partial separation of α2,6- and α2,3-sialylated PSA isomers. Besides the real potential of this approach should be evaluated by the analysis of clinical samples. Altered sialylation and glycosylation content of PSA were also investigated in prostate cancer and patient-matched non-cancer tissues by Li et al. They developed an SRM assay to quantify formerly glycosylated and sialylated PSA [73]. In this approach, glycopeptides were isolated from tryptic digests of tissue samples (PCa = 9 and non-PCa = 9 obtained from the same 9 subjects) by either SPEG or by a modified SPEG protocol able to capture sialylated glycopeptides by using mild oxidation conditions. Quantification of formerly N-glycosylated peptides obtained from the two parallel enrichment protocols, carried out by SRM, was performed using a heavy-isotope-labelled PSA peptide. Results showed that there was no correlation between total PSA (measured by immunoassay) and the abundance of either glycosylated or sialylated PSA. Besides, no significant differences were observed in either total PSA glycosylation or sialylation between PCa and non-PCa groups.

Chun-Jen Hsiao et al. quantified the relative abundance of urinary PSA glycoforms in BPH and PCa patients [74]. In particular, PSA was captured from 50 mL of urine using anti-PSA beads, denatured, separated by SDS-PAGE and finally digested with chymotrypsin and analysed by LC-MS/MS. PSA glycoforms were quantified by a label-free method; the abundance of each PSA glycopeptide was normalized using the following equation:

Level of PSA glycopeptide = (Glycopeptide ion abundance)/(PSA protein internal reference peptide ion abundance) × 100.

The authors found that the two most frequently observed glycoforms in BPH samples were H5N4S1F1 (71%, H: hexose; N: N-acetylhexosamine; S: sialic acid; F: fucose) and H6N3S1 (69%) while in PCa were H5N4S2F1 and H6N3S1 (55%). Though this work represents the first attempt to exploit the detection of specific PSA glycoforms for clinical purposes, it shows several important limitations regarding sample collection and analysis. Notably, no internal reference peptide or PSA glycopeptide could be detected in almost one third of the sample set (originally comprising 61 PBH and 38 PCa samples), determining the exclusion of these samples from subsequent data analysis. By considering either relative levels of total glycopeptides or of unfucosylated glycoforms, the authors achieved a sensitivity of 88% and a specificity of 60% in classifying the remaining samples (43 BPH and 20 PCa).

In an attempt to improve the efficiency of PSA in discriminating PCa from BPH and AG PCa from NAG PCa, Lang et al., investigated PSA-core fucosylation [61]. Patients enrolled in the study were 150 (BPH = 50, AG-PCa = 50 and NAG-PCa = 50), all within the PSA diagnostic grey zone. The quantification of total and core-fucosylated PSA was obtained by immunoaffinity enrichment of PSA from serum, partial deglycosylation by Endo F3 (specific cleavage within the chitobiose core of N-linked fucosylated-biantennary and triantennary oligosaccharides), followed by tryptic digestion and then LC-MS/MS analysis. Finally, total PSA and fuc-PSA were analysed by measuring peptide LSEPAELTDAVK and glycopeptide NK + GlcNAc + Fuc, respectively. The data showed that total PSA concentrations of samples measured by LC-MS/MS and electrochemiluminescence immunoassay analyser (ECLIA) exhibited a good agreement. However, %-Free PSA (AUC of 0.74) outperformed fuc-PSA (AUC 0.58) in correctly classifying BPH and PCa samples. Moreover, fuc-PSA was not able to better stratify aggressive and non-aggressive PCa in comparison to standard methods (%-Free PSA and total PSA). Glycans of PSA were also investigated in PCa tissue-originated spheroids (CTOS) [75]. The cardinal principle of this study was that CTOS-derived PSA is considered to reflect glycan structures of the patient’s tumour. PSA glycan profile obtained from a single patient’s CTOS was compared with that of PSA from normal seminal plasma and two cancer cell lines (LNCaP and 22Rv1) using lectin chromatography and mass spectrometry. The results indicated a higher fraction of (Concanavalin A)-unbound PSA from the three cancer cell types with respect to seminal plasma. Interestingly, in the Con A-unbound fraction, 2 novel forms of PSA were identified: a high molecular weight PSA with highly branched N-glycans and a low molecular weight PSA which was either a truncated or an unglycosylated isoform. Being common to all samples of cancer origin and not to seminal PSA, the two novel glycoforms are proposed as potential new cancer markers which will be the object of follow-up studies.

The most promising MS-based study on PSA glycosylation is perhaps that of Haga et al. This study established a novel PCa-specific diagnostic model (PSA G-index) integrating immuno-affinity enrichment of PSA from serum and mass spectrometric oxonium ion monitoring of tryptic peptides [76]. PSA glycoforms were quantitatively evaluated in sera from 15 PCa or 15 BPH patients having PSA levels in the diagnostic grey zone. The data showed that abundances of di/trisialylated LacdiNAc (GalNAcβ1-4GlcNAc)-containing structures were significantly increased in the PCa group compared to the BPH group. In total, 52 glycan structures were quantified using 100 µL of serum as starting material. Then, PSA G-index was devised using 2 of these glycoforms and was tested in an independent sample cohort (15 PCA vs. 15 BPH falling in the diagnostic grey zone). In this limited sample set, the AUC of PSA G-index was 1, while that of total PSA or %freePSA was 0.50 or 0.60, respectively. Moreover, both PSA glycoforms showed significant correlation with Gleason scores. Thus, PSA G-index could drastically improve the specificity of PCa diagnosis compared to traditional blood testing but large-scale experiments are needed to confirm these preliminary results.

Some of the most relevant findings obtained in the comparative analyses described and depicted in this paragraph are summarized in Table 5.

## 5. Conclusions

The results reported in this review demonstrate that glycoproteomics analysis on cell lines or tissues is helping in further clarifying the molecular dynamics around PCa onset and development. For example, several studies mentioned above have reported altered fucosylation in cancer cells and have linked such alterations to differential expression of fucosyltransferases like FUT8; this was the starting point for functional validation experiments demonstrating the involvement of FUT8 in integrin-mediated cell migration and signalling and in stabilizing EGFR in cancer cells.

Concerning biofluid analysis, despite the identification of several novel candidate biomarkers, glycoproteomics has not yet identified an analyte or a protein panel able to replace PSA in the clinical practice. However, the analysis of PSA glycoforms is promising, though it requires further validation studies in order to assess its real advantages over the mere dosage of free and total PSA. Although, to date, glycoproteomic analysis of urine has been performed only at an initial discovery level, since this sample is a prostate proximal fluid, it holds promise for future success in PCa early detection.

Although the ultimate goal of many of the studies discussed in this review is the introduction in the clinical practice of a glycoprotein or a panel of glycoproteins able to improve PCa diagnosis, the achievement of this aim is still a long way off. In general, in fact, the best candidates detected in the discovery phase must be validated through the recruitment of large cohorts of patients, involving several laboratories. However, the realization of such multicentric studies is hindered by some elements such as the elevated costs of experiments, the complexity and laboriousness of extraction and analysis protocols currently in use and the absence of standards to perform the absolute quantitation of glycan-bearing peptides. Moreover, the commonly used label-free approach holds some intrinsic limitations, mainly represented by its limited robustness and precision. Thus, the improvement of these methodological aspects will be of pivotal importance to allow the implementation of large-scale studies and, consequently, the introduction in the clinical routine of the validated biomarkers.

Making assumptions about the future, we could hypothesize that, the achievement of more standardized protocols (sample collection, processing and analysis) will allow a more prominent contribute of MS to clinical practice.

## Figures and Tables

**Table 1:** Main differentially abundant glycoproteins (DAPs) identified in cell cultures or PCa tissue by MS-based workflows. Plain text = discovery level; bold character = validation level; italic = protein is downregulated; (*f*) core-fucosylated glycopeptide; (*g*) glycopeptide.

Ref.	Sample Groups	Workflow	Analyte	Main DAPs
[29]	Cells: LNCaP and PC3	SPEG	N-glycosites, glycopeptides	*N-glycosite analysis*: FBN3, CD55, NT5E, MRC2, SIDT1, TICAM2, ICAM1, TSPAN3, PLOD2, CD44 *glycopeptide analysis*: APMAP (*f*), PSAP (*f*), HSP90B1 (*f*), CD63 (*g*)
[31]	Cells: LNCaP/LAPC4 (NAG) vs. PC3/DU145 (AG)	LAC- HILIC	Glycopeptides	NT5E (*f*), CALU (*f*), CPM (*f*), HYOU1 (*f*), ITGB1 (*f*), ITGA2 (*f*), ITGA3 (*f*), PSAP (*f*), CD40 (*f*), EGFR (*f*), ASPH (*f*)
[36]	Cells: nonmetastatic (N2) and highly metastatic (ML2) PC3	Met. Lab./SPE-SG	Sialo- glycoproteins	BSG, POSTN, GPI, CALR, LRRC15, MARCKS, JUP, PTPN1
[35]	Tissue: PCa versus normal	Met. Lab./SPE-SG	Sialo- glycoproteins	VDAC1, DPP2, EZRI, GDF15, FOLH, CATB, AMPN
[38]	Tissue: NAG vs. AG	SPEG	N-glycosites	**COMP**, **POSTN**, ***CSPG2***, AOC3, CTSL, APMAP, EMILIN-3
[40]	Tissue: N, NAG, AG, MET	SPEG	N-glycosites	AG vs. NAG: POSTN, ASPN, LAMB2, SERPINH1, CSPG2, ENTPD1, SEL1L, ITGAV, FOLH1, STIM1, **PTK7**, ICOSLG, SPPL2A, CD276, ***NAAA***, *CNTNAP2*, *AMPN* NAG/AG vs. N: CTSD, ATRN, BTD, MTA1
[42]	Tissue: PCa vs. BPH	ZIC-HILIC	Intact glycopeptides, N-glycosites, glycans	*Glycans analysis* Low grade PCa vs. BPH: paucimannosidic- and monoantennary-complex. High grade PCa vs. BPH: highly branched complex, *oligomannosidic-*, *hybrid-* and *biantennary complex*.
[43]	Cells: M2 vs. M1 macrophages	SPEG	N-glycosites	MRC1, CTSL, ITGA3, LGMN, SLC9A7

**Table 2:** AUC values obtained by different combinations of candidate biomarkers in the discovery and validation cohorts [49].

Panel of Candidate Biomarkers	Area under the ROC Curve (95% Confidence Interval)		
	Discovery cohort	Validation cohort	Validation cohort
		40 AG and 37 NAG (set 1)	40 AG and 13 NAG (set2)
ACPP & Serum PSA	0.82 (0.75, 0.89)	0.83 (0.74, 0.92)	0.8 (0.67, 0.93)
ACPP & CLU & Serum PSA	0.86 (0.8, 0.92)	0.85 (0.76, 0.94)	0.76 (0.6, 0.92)
ACPP & LOX & Serum PSA	0.82 (0.75, 0.89)	0.85 (0.76, 0.93)	0.81 (0.69, 0.93)
ACPP & SERPINA1 & Serum PSA	0.83 (0.76, 0.9)	0.84 (0.75, 0.93)	0.82 (0.7, 0.94)
ACPP & ORM1 & Serum PSA	0.83 (0.76, 0.9)	0.82 (0.72, 0.91)	0.82 (0.71, 0.94)

**Table 3:** Main differentially abundant glycoproteins (DAPs) identified in PCa urine samples by MS-based workflows. Plain text = discovery level; bold character = validation level; italic = protein is downregulated.

Reference	Sample	Sample Groups	Workflow	Analyte	Main DAPs
[46]	Urine	AG vs. NAG	SPEG	N-glycosites	*PTK7*, *ICOSL*, *AZGP1*, *FBN1*, *GLG1*
[47]	Urine	PCa vs. BPH	TiO_2_ and HILIC	Intact glycopeptides, N-glycosites, de-sialo-O-glycopeptides	*Intact N-* *glycopeptides analysis*: AMBP, CD59, CLU, LOX
[49]	EPS-urine	AG vs. NAG	C18/MAX tips	N-glycosites	***ACPP***, *CD63*, DSC2, **LOX**, LRG1, **CLU**, **SERPINA1**, **ORM1**

**Table 4:** Main differentially abundant glycoproteins (DAPs) identified in PCa urine sample by MS-based workflows. Plain text = discovery level; bold character = validation level; italic = protein is downregulated; (*f*) core-fucosylated glycopeptide; (*g*) glycopeptide, (*h*) highly branched glycopeptide.

Reference	Sample	Sample Groups	Workflow	Analyte	Main DAPs
[41]	Serum	PCA vs. BPH	SPEG	N-glycosites	**ASPN**, **CTSD**, **HYOU1**, **OLFM4**, AZGP1, POSTN
[52]	Serum	mCRPC	SPEG	N-glycosites	**THBS1**, **CRP**, **PVLR1**, **EFNA5**, **MME**, AZGP1, POSTN
[53]	Serum	NAG vs. AG	SPEG	N-glycosites	CD44, IGHG2, ITIH2, CDH13
[54]	Serum	PCa vs. BPH	M-LAC	glycoproteins	CD5L, CFP, C8A, BST1, C7 CD163 (*h*), C4A (*h*) ATR (*h*), C4BPB (*f*) and AZGP1 (*f*)
[55]	Serum	colorectal, pancreatic, lung, prostate, and ovarian cancer	SPEG	N-glycosites	Pancancer candidate biomarkers: FBN1, THBS1, ITIH3; PCa specific candidate biomarker: **PZP**
[56]	Serum	PCa vs. BPH	TiO_2_	N-glycosites	APMAP, POSTN, CATD and LAMP2

**Table 5:** Main differentially abundant glycoforms of PSA found increased in PCa. * indicates non-MS-based workflows. Plain text = discovery level; bold character = validation level. Bead capture = PSA immunopurification by anti-PSA microbeads.

Ref.	Sample	Sample Groups	Workflow	PSA Glycoforms	Depiction
[68]	Serum	Non-PCa = 176 and PCa = 138	Bead-capture *	**α2,3-sialic acid-linked**	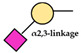
[69]	Serum	BPH= 29 and low-risk PCa = 7, intermediate risk PCa = 21, high-risk PCa = 22	Bead-capture and lectin chromatography *	α2,3-sialic acid-linked (in high risk PCa)	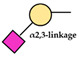
[74]	Urine	BPH = 43 and PCa = 20	Bead-capture	H5N4S2F1 and H6N3S1 glycoforms	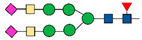 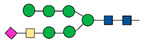
[76]	Serum	PCA = 15 and BPH = 15	Bead-capture	di/trisialylated (GalNAcβ1-4GlcNAc)-containing structures	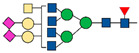 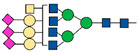

## Data Availability

Data sharing not applicable.

## References

[1] Siegel R.L., Miller K.D. (2021). Cancer Statisics, 2021. CA Cancer J. Clin..

[2] Thompson I.M., Pauler D.K., Goodman P.J., Tangen C.M., Lucia M.S., Parnes H.L., Minasian L.M., Ford L.G., Lippman S.M., Crawford E.D. (2004). Prevalence of prostate cancer among men with a prostate-specific antigen level ≤ 4.0 ng per milliliter. N. Engl. J. Med..

[3] Lumen N., Fonteyne V., De Meerleert G., Ost P., Villeirs G., Mottrie A., De Visschere P., De Troyer B., Oosterlinck W. (2012). Population screening for prostate cancer: An overview of available studies and meta-analysis. Int. J. Urol..

[4] Pin E., Fredolini C., Petricoin E.F. (2013). The role of proteomics in prostate cancer research: Biomarker discovery and validation. Clin. Biochem..

[5] Olsen J.V., Mann M. (2013). Status of large-scale analysis of post-translational modifications by mass spectrometry. Mol. Cell. Proteom. MCP.

[6] Moremen K.W., Tiemeyer M., Nairn A.V. (2012). Vertebrate protein glycosylation: Diversity, synthesis and function. Nat. Rev. Mol. Cell Biol..

[7] Schachter H., Freeze H.H. (2009). Glycosylation diseases: Quo vadis?. Biochim. Et Biophys. Acta Mol. Basis Dis..

[8] Cummings R.D. (2009). The repertoire of glycan determinants in the human glycome. Mol. Biosyst..

[9] Spiro R.G. (2002). Protein glycosylation: Nature, distribution, enzymatic formation, and disease implications of glycopeptide bonds. Glycobiology.

[10] Drake P.M., Cho W., Li B., Prakobphol A., Johansen E., Anderson N.L., Regnier F.E., Gibson B.W., Fisher S.J. (2010). Sweetening the Pot: Adding Glycosylation to the Biomarker Discovery Equation. Clin. Chem..

[11] Munkley J., Elliott D.J. (2016). Hallmarks of glycosylation in cancer. Oncotarget.

[12] Peracaula R., Barrabés S., Sarrats A., Rudd P.M., de Llorens R. (2008). Altered Glycosylation in Tumours Focused to Cancer Diagnosis.

[13] Munkley J., Mills I.G., Elliott D.J. (2016). The role of glycans in the development and progression of prostate cancer. Nat. Rev. Urol..

[14] Dwek M.V., Jenks A., Leathem A.J.C. (2010). A sensitive assay to measure biomarker glycosylation demonstrates increased fucosylation of prostate specific antigen (PSA) in patients with prostate cancer compared with benign prostatic hyperplasia. Clin. Chim. Acta.

[15] Peracaula R., Tabarés G., Royle L., Harvey D.J., Dwek R.A., Rudd P.M., de Llorens R. (2003). Altered glycosylation pattern allows the distinction between prostate-specific antigen (PSA) from normal and tumor origins. Glycobiology.

[16] Li J., Guillebon A.D., Hsu J.-W., Barthel S.R., Dimitroff C.J., Lee Y.-F., King M.R. (2013). Human fucosyltransferase 6 enables prostate cancer metastasis to bone. Br. J. Cancer.

[17] Kolli V., Schumacher K.N., Dodds E.D. (2015). Engaging challenges in glycoproteomics: Recent advances in MS-based glycopeptide analysis. Bioanalysis.

[18] Schirm M., Schoenhofen I.C., Logan S.M., Waldron K.C., Thibault P. (2005). Identification of unusual bacterial glycosylation by tandem mass spectrometry analyses of intact proteins. Anal. Chem..

[19] De Leoz M.L.A., An H.Y., Kronewitter S., Kim J., Beecroft S., Vinall R., Miyamoto S., de Vere White R., Lam K.S., Lebrilla C. (2008). Glycomic approach for potential biomarkers on prostate cancer: Profiling of N-linked glycans in human sera and pRNS cell lines. Dis. Markers.

[20] Dai L., Liu Y., He J., Flack C.G., Talsma C.E., Crowley J.G., Muraszko K.M., Fan X., Lubman D.M. (2011). Differential profiling studies of N-linked glycoproteins in glioblastoma cancer stem cells upon treatment with γ-secretase inhibitor. Proteomics.

[21] Cummings R., Kornfeld S. (1982). Fractionation of asparagine-linked oligosaccharides by serial lectin-agarose affinity chromatography. J. Biol. Chem.

[22] Hirabayashi J. (2004). Lectin-based structural glycomics: Glycoproteomics and glycan profiling. Glycoconj. J..

[23] Zhang H., Li X.J., Martin D.B., Aebersold R. (2003). Identification and quantification of N-linked glycoproteins using hydrazide chemistry, stable isotope labeling and mass spectrometry. Nat. Biotechnol..

[24] Larsen M.R., Jensen S.S., Jakobsen L.A., Heegaard N.H.H. (2007). Exploring the sialiome using titanium dioxide chromatography and mass spectrometry. Mol. Cell. Proteom..

[25] Mahal L.K., Yarema K.J., Bertozzi C.R. (1997). Engineering chemical reactivity on cell surfaces through oligosaccharide biosynthesis. Science.

[26] Bond M.R., Kohler M.J. (2007). Chemical methods for glycoprotein discovery. Curr. Opin. Chem. Biol..

[27] Campbell M.P., Packer N.H. (2016). UniCarbKB: New database features for integrating glycan structure abundance, compositional glycoproteomics data, and disease associations. Biochim. Et Biophys. Acta Gen. Subj..

[28] Varki A., Cummings R.D., Esko J.D., Stanley P., Hart G.W., Aebi M., Darvill A.G., Kinoshita T., Packer N.H., Prestegard J.H. (2015). Future Directions in Glycosciences. Essentials of Glycobiology.

[29] Shah P., Wang X., Yang W., Eshghi S.T., Sun S., Hoti N., Chen L., Yang S., Pasay J., Rubin A. (2015). Integrated Proteomic and Glycoproteomic Analyses of Prostate Cancer Cells Reveal Glycoprotein Alteration in Protein Abundance and Glycosylation. Mol. Cell. Proteom..

[30] Wang X., Chen J., Li Q.L., Peskoe S.B., Zhang B., Choi C., Platz A., Zhang H. (2014). Overexpression of α (1,6) fucosyltransferase associated with aggressive prostate cancer. Glycobiology.

[31] Zhou J., Yang W., Hu Y., Höti N., Liu Y., Shah P., Sun S., Clark D., Thomas S., Zhang H. (2017). Site-Specific Fucosylation Analysis Identifying Glycoproteins Associated with Aggressive Prostate Cancer Cell Lines Using Tandem Affinity Enrichments of Intact Glycopeptides Followed by Mass Spectrometry. Anal. Chem..

[32] Höti N., Lih T.-S., Pan J., Zhou Y., Yang G., Deng A., Chen L., Dong M., Yang R.-B., Tu C.-F. (2020). A comprehensive analysis of FUT8 overexpressing prostate cancer cells reveals the role of EGFR in castration resistance. Cancers.

[33] Rodrigues E., Macauley M.S. (2018). Hypersialylation in cancer: Modulation of inflammation and therapeutic opportunities. Cancers.

[34] Hubbard S.C., Boyce M., McVaugh C.T., Peehl D.M., Bertozzi C.R. (2011). Cell surface glycoproteomic analysis of prostate cancer-derived PC-3 cells. Bioorg. Med. Chem. Lett..

[35] Spiciarich D.R., Nolley R., Maund S.L., Purcell S.C., Herschel J., Iavarone A.T., Peehl D.M., Bertozzi C.B. (2017). Bioorthogonal Labeling of Human Prostate Cancer Tissue Slice Cultures for Glycoproteomics. Angew. Chem. Int. Ed..

[36] Yang L., Nyalwidhe J.O., Guo S., Drake R.R., Semmes O.J. (2011). Targeted Identification of Metastasis-associated Cell-surface Sialoglycoproteins in Prostate Cancer. Mol. Cell. Proteom. MCP.

[37] Arif T., Vasilkovsky L., Refaely Y., Konson A., Shoshan-Barmatz V. (2014). Silencing VDAC1 Expression by siRNA Inhibits Cancer Cell Proliferation and Tumor Growth In Vivo. Mol. Ther. Nucleic Acids.

[38] Chen J., Xi J., Tian Y., Bova G.S., Zhang H. (2013). Identification, prioritization, and evaluation of glycoproteins for aggressive prostate cancer using quantitative glycoproteomics and antibody-based assays on tissue specimens. Proteomics.

[39] Tian Y., Bova G.S., Zhang H. (2011). Quantitative Glycoproteomic Analysis of Optimal Cutting Temperature-Embedded Frozen Tissues Identifying Glycoproteins Associated with Aggressive Prostate Cancer. Anal. Chem..

[40] Liu Y., Chen J., Sethi A., Li Q.K., Chen L., Collins B., Gillet L.C.J., Wollscheid B., Zhang H., Aebersold R. (2014). Glycoproteomic Analysis of Prostate Cancer Tissues by SWATH Mass Spectrometry Discovers N-acylethanolamine Acid Amidase and Protein Tyrosine Kinase 7 as Signatures for Tumor Aggressiveness. Mol. Cell. Proteom. MCP.

[41] Cima I., Schiess R., Wild P., Kaelin M., Schüffler P., Lange V., Picotti P., Ossola R., Templeton A., Schubert O. (2011). Cancer genetics-guided discovery of serum biomarker signatures for diagnosis and prognosis of prostate cancer. Proc. Natl. Acad. Sci. USA.

[42] Kawahara R., Recuero S., Srougi M., Leite K.R.M., Thaysen-Andersen M., Palmisano G. (2020). The complexity and dynamics of the tissue glycoproteome associated with prostate cancer progression. Mol. Cell. Proteom..

[43] Zarif J.C., Yang W., Hernandez J.R., Zhang H., Pienta K.J. (2017). The Identification of Macrophage-enriched Glycoproteins Using Glycoproteomics. Mol. Cell. Proteom..

[44] Haj-ahmad T.A., Abdalla M.A.K., Haj-Ahmad Y. (2014). Potential Urinary Protein Biomarker Candidates for the Accurate Detection of Prostate Cancer among Benign Prostatic Hyperplasia Patients. J. Cancer.

[45] Kawahara R., Saad J., Angeli C.B., Palmisano G. (2016). Site-specific characterization of N-linked glycosylation in human urinary glycoproteins and endogenous glycopeptides. Glycoconj. J..

[46] Jia X., Chen J., Sun S., Yang W., Yang S., Shah P., Hoti N., Veltri B., Zhang H. (2016). Detection of aggressive prostate cancer associated glycoproteins in urine using glycoproteomics and mass spectrometry. Proteomics.

[47] Kawahara R., Ortega F., Rosa-Fernandes L., Guimarães V., Quina D., Nahas W., Schwämmle V., Srougi M., Leite K.R.M., Thaysen-Andersen M. (2018). Distinct urinary glycoprotein signatures in prostate cancer patients. Oncotarget.

[48] Vermassen T., Van Praet C., Vanderschaeghe D., Maenhout T., Lumen N., Callewaert N., Hoebeke P., Van Belle S., Rottey S., Delanghe J. (2014). Capillary electrophoresis of urinary prostate glycoproteins assists in the diagnosis of prostate cancer. Electrophoresis.

[49] Dong M., Lih T.M., Chen S.Y., Cho K.C., Eguez R.V., Höti N., Zhou Y., Yang W., Mangold L., Chan D.W. (2020). Urinary glycoproteins associated with aggressive prostate cancer. Theranostics.

[50] Clark D.J., Hu Y., Schnaubelt M., Fu Y., Ponce S., Chen S.Y., Zhou Y., Shah P., Zhang H. (2019). Simple Tip-Based Sample Processing Method for Urinary Proteomic Analysis. Anal. Chem..

[51] Chen S.-Y., Dong M., Yang G., Zhou Y., Clark D.J., Lih T.M., Schnaubelt M., Liu Z., Zhang H. (2020). Glycans, Glycosite, and Intact Glycopeptide Analysis of N-Linked Glycoproteins Using Liquid Handling Systems. Anal. Chem..

[52] Kälin M., Cima I., Schiess R., Fankhauser N., Powles T., Wild P., Templeton A., Cerny T., Aebersold R., Krek W. (2011). Novel Prognostic Markers in the Serum of Patients with Castration-Resistant Prostate Cancer Derived from Quantitative Analysis of the Pten Conditional Knockout Mouse Proteome. Eur. Urol..

[53] Thomas S.N., Harlan R., Chen J., Aiyetan P., Liu Y., Sokoll L.J., Aebersold R., Chan D.W., Zhang H. (2015). Multiplexed Targeted Mass Spectrometry-Based Assays for the Quantification of N-Linked Glycosite-Containing Peptides in Serum. Anal. Chem..

[54] Totten S.M., Adusumilli R., Kullolli M., Tanimoto C., Brooks J.D., Mallick P., Pitteri S.J. (2018). Multi-lectin Affinity Chromatography and Quantitative Proteomic Analysis Reveal Differential Glycoform Levels between Prostate Cancer and Benign Prostatic Hyperplasia Sera. Sci. Rep..

[55] Sajic T., Liu Y., Arvaniti E., Surinova S., Williams E.G., Schiess R., Hüttenhain R., Sethi A., Pan S., Brentnall T.A. (2018). Similarities and Differences of Blood N-Glycoproteins in Five Solid Carcinomas at Localized Clinical Stage Analyzed by SWATH-MS’. Cell Rep..

[56] Gabriele C., Cantiello F., Nicastri A., Crocerossa F., Russo G.I., Cicione A., Vartolomei M.D., Ferro M., Morgia G., Lucarelli G. (2019). High-throughput detection of low abundance sialylated glycoproteins in human serum by TiO_2_ enrichment and targeted LC-MS/MS analysis: Application to a prostate cancer sample set. Anal. Bioanal. Chem..

[57] Balk S.P., Ko Y.J., Bubley G.J. (2003). Biology of prostate-specific antigen. J. Clin. Oncol..

[58] Moradi A., Srinivasan S., Clements J., Batra J. (2019). Beyond the biomarker role: Prostate-specific antigen (PSA) in the prostate cancer microenvironment. Cancer Metastasis Rev..

[59] Kulasingam V., Diamandis E.P. (2008). Strategies for discovering novel cancer biomarkers through utilization of emerging technologies. Nat. Clin. Pract. Oncol..

[60] Prensner J.R., Rubin M.A., Wei J.T., Chinnaiyan A.M. (2012). Beyond PSA: The next generation of prostate cancer biomarkers. Sci. Transl. Med..

[61] Lang R., Rolny V., Leinenbach A., Karl J., Swiatek-de Lange M., Kobold U., Schrader M., Krause H., Mueller M., Vogeser M. (2019). Investigation on core-fucosylated prostate-specific antigen as a refined biomarker for differentiation of benign prostate hyperplasia and prostate cancer of different aggressiveness. Tumor Biol..

[62] Neal D.E., Donovan J.L., Martin R.M., Hamdy F.C. (2009). Screening for prostate cancer remains controversial. Lancet.

[63] Reider B., Jarvas G., Krenkova J., Guttman A. (2021). Separation based characterization methods for the N-glycosylation analysis of prostate-specific antigen. J. Pharm. Biomed. Anal..

[64] Wang W., Kałuża A., Nouta J., Nicolardi S., Ferens-Sieczkowska M., Wuhrer M., Lageveen-Kammeijer G.S.M., de Haan N. (2021). High-throughput glycopeptide profiling of prostate-specific antigen from seminal plasma by MALDI-MS. Talanta.

[65] Leymarie N., Griffin P.J., Jonscher K., Kolarich D., Orlando R., McComb M., Zaia J., Aguilan J., Alley W.R., Altmann F. (2013). Interlaboratory Study on Differential Analysis of Protein Glycosylation by Mass Spectrometry: The ABRF Glycoprotein Research Multi-Institutional Study 2012. Mol. Cell. Proteom..

[66] Behnken H.N., Ruthenbeck A., Schulz J.-M., Meyer B. (2014). Glycan analysis of Prostate Specific Antigen (PSA) directly from the intact glycoprotein by HR-ESI/TOF-MS. J. Proteome Res..

[67] Song E., Hu Y., Yu C.-Y., Tang H., Mechref Y. (2016). Comprehensive Characterization of the Glycosylation Site of Human PSA Prompted by Missense Mutation using LC-MS/MS. J. Proteome Res..

[68] Yoneyama T., Ohyama C., Hatakeyama S., Narita S., Habuchi T., Koie T., Mori K., Hidari K.I.P.J., Yamaguchi M., Suzuki T. (2014). Measurement of aberrant glycosylation of prostate specific antigen can improve specificity in early detection of prostate cancer. Biochem. Biophys. Res. Commun..

[69] Ferrer-Batallé M., Llop E., Ramírez M., Aleixandre R.N., Saez M., Come J., de Llorens R., Peracaula R. (2017). Comparative study of blood-based biomarkers, α2,3-sialic acid PSA and PHI, for high-risk prostate cancer detection. Int. J. Mol. Sci..

[70] Kammeijer G.S.M., Jansen B.C., Kohler I., Heemskerk A.A.M., Mayboroda O.A., Hensbergen P.J., Schappler J., Wuhrer M. (2017). Sialic acid linkage differentiation of glycopeptides using capillary electrophoresis-Electrospray ionization-Mass spectrometry. Sci. Rep..

[71] Kammeijer G.S.M., Nouta J., de la Rosette J.J.M.C.H., de Reijke T.M., Wuhrer M. (2018). An In-Depth Glycosylation Assay for Urinary Prostate-Specific Antigen. Anal. Chem..

[72] van der Burgt Y.E.M., Siliakus K.M., Cobbaert C.M., Ruhaak L.R. (2020). HILIC–MRM–MS for Linkage-Specific Separation of Sialylated Glycopeptides to Quantify Prostate-Specific Antigen Proteoforms. J. Proteome Res..

[73] Li Y., Tian Y., Rezai T., Prakash A., Lopez M.F., Chan D.W., Zhang H. (2011). Simultaneous Analysis of Glycosylated and Sialylated Prostate-Specific Antigen Revealing Differential Distribution of Glycosylated Prostate-Specific Antigen Isoforms in Prostate Cancer Tissues. Anal. Chem..

[74] Yang W.-H., Chen C.-H., Tzai T.-S., Chen C.-H., Hsiao C.-J. (2016). Analysis of Urinary Prostate-Specific Antigen Glycoforms in Samples of Prostate Cancer and Benign Prostate Hyperplasia. Dis. Markers.

[75] Ideo H., Kondo J., Nomura T., Nonomura N., Inoue M., Amano J. (2020). Study of glycosylation of prostate-specific antigen secreted by cancer tissue-originated spheroids reveals new candidates for prostate cancer detection. Sci. Rep..

[76] Haga Y., Uemura M., Baba S., Inamura K., Takeuchi K., Nonomura N., Ueda K. (2019). Identification of Multisialylated LacdiNAc Structures as Highly Prostate Cancer Specific Glycan Signatures on PSA. Anal. Chem..

